# A Recommendation System for Prosumers Based on Large Language Models

**DOI:** 10.3390/s24113530

**Published:** 2024-05-30

**Authors:** Simona-Vasilica Oprea, Adela Bâra

**Affiliations:** Department of Economic Informatics and Cybernetics, Bucharest University of Economic Studies, No. 6 Piaţa Romană, 010374 Bucharest, Romania; bara.adela@ie.ase.ro

**Keywords:** LLMs, recommendation system, prosumers, energy communities, RES integration

## Abstract

As modern technologies, particularly home assistant devices and sensors, become more integrated into our daily lives, they are also making their way into the domain of energy management within our homes. Homeowners, now acting as prosumers, have access to detailed information at 15-min or even 5-min intervals, including weather forecasts, outputs from renewable energy source (RES)-based systems, appliance schedules and the current energy balance, which details any deficits or surpluses along with their quantities and the predicted prices on the local energy market (LEM). The goal for these prosumers is to reduce costs while ensuring their home’s comfort levels are maintained. However, given the complexity and the rapid decision-making required in managing this information, the need for a supportive system is evident. This is particularly true given the routine nature of these decisions, highlighting the potential for a system that provides personalized recommendations to optimize energy consumption, whether that involves adjusting the load or engaging in transactions with the LEM. In this context, we propose a recommendation system powered by large language models (LLMs), Scikit-llm and zero-shot classifiers, designed to evaluate specific scenarios and offer tailored advice for prosumers based on the available data at any given moment. Two scenarios for a prosumer of 5.9 kW are assessed using candidate labels, such as Decrease, Increase, Sell and Buy. A comparison with a content-based filtering system is provided considering the performance metrics that are relevant for prosumers.

## 1. Introduction

Prosumers often face significant challenges in aligning their energy generation with their daily consumption schedules. This issue is particularly critical for off-grid prosumers, as any unused energy is wasted. Additionally, photovoltaic (PV) systems, which are prone to significant fluctuations, especially during the cold season, add to the difficulty of efficiently consuming the energy they generate [1]. Another major challenge involves managing the integration of generating systems, smart appliances, heating, and other household systems. Data from various sources, including occupancy and temperature sensors, PV systems, weather data, and forecasting applications, need to be correlated. Effective actions must then be taken based on this data to reduce electricity consumption costs.

Home energy management systems (HEMSs) have become increasingly sophisticated, integrating various types of sensors and smart assistants to optimize energy usage for prosumers. They are designed to monitor and control energy usage in homes. These systems often include smart meters to measure electricity consumption in real-time, energy dashboards to provide homeowners with insights into their energy use, and automated controls that adjust heating, cooling, and lighting based on pre-set preferences or real-time data [2]. Home assistants such as Amazon Alexa, Google Home, and Apple HomePod can be integrated into HEMSs to facilitate voice control and automation. These assistants can control appliances and lighting, providing users with the ability to turn devices on or off based on voice commands or routines. They also offer energy reports, informing users about their energy consumption patterns and implementing energy-saving modes by adjusting settings based on occupancy or time of day to save energy [3].

Presence sensors detect the presence of individuals in various rooms, automating lighting and HVAC systems to turn lights on or off and adjust heating or cooling based on room occupancy. They enhance security by alerting homeowners of unexpected movements when the house is supposed to be empty and optimize energy usage by ensuring energy is used only when and where it is needed. Temperature sensors monitor the ambient temperature in different areas of the home and regulate HVAC systems to maintain optimal temperatures in occupied areas, reducing heating or cooling in unoccupied areas [4]. They provide data for energy management, helping HEMS adjust settings for energy efficiency, and integrate with smart thermostats like Nest or Ecobee, which use temperature sensors to learn homeowner preferences and optimize heating or cooling schedules.

Integrating these sensors into recommender systems is important to fully leverage the data they provide. Recommender systems analyze data from various sensors and offer actionable recommendations to prosumers, such as energy-saving suggestions for thermostat settings, appliance usage or lighting schedules. They also provide maintenance alerts, notifying users when appliances are operating inefficiently or need servicing, and offer insights into PV forecasting based on sensors [5], usage patterns to help prosumers understand their energy consumption and identify opportunities for savings.

An example of implementation involves data collection from sensors throughout the home, which is then processed by the HEMS to understand patterns and detect anomalies. The system provides recommendations via the home assistant, such as lowering the thermostat when the house is empty or suggesting off-peak times for running energy-intensive appliances. Based on user preferences and historical data, the HEMS can automate energy-saving actions like dimming lights or adjusting HVAC settings without manual intervention. The benefits for prosumers include cost savings by optimizing energy usage, which significantly reduces energy bills. Efficient energy management also reduces the overall carbon footprint of the household [6,7], offering environmental benefits. These recommendations empower users to make informed decisions about their energy use.

Recommendation systems for prosumers in the context of energy management involve sophisticated algorithms designed to analyze various data points, like energy production, consumption patterns, weather forecasts and market prices, even social media insights and news [8] to provide personalized advice. These systems aim to optimize energy use, reduce costs and even generate revenue by engaging in energy trading [9]. General types of recommendation systems and technologies that play a significant role for prosumers in the energy sector are usually associated with the following: (a) smart home energy management systems or HEMSs, systems which integrate with smart home devices and RESs (like solar panels) to optimize energy consumption [10] and which may suggest the best times to use energy-intensive appliances based on the lowest energy prices or highest RES production, contributing to cost savings and increased energy efficiency [11,12]; (b) demand–response (DR) programs, which are often run by utilities to incentivize prosumers to reduce or shift their energy consumption during peak intervals [13,14] and which include recommendation systems that may alert users to these opportunities, advising when to decrease usage or temporarily store energy in home batteries for later use or sale back to the grid; (c) predictive analytics for RES production, which forecast production levels based on weather data and historical performance and which may recommend optimal times to store, use or sell energy, maximizing the financial and environmental benefits of RES installations [15]; (d) peer-to-peer (P2P) energy trading platforms, through which emerging blockchain and digital platform technologies enable P2P energy trading, allowing prosumers to sell excess renewable energy directly to neighbors or other local consumers [16,17] and which may suggest the best times to buy or sell energy based on market trends and individual consumption patterns; (e) LLMs and other artificial intelligence (AI) technologies for personalized recommendations, which may analyze vast datasets to provide tailored advice for energy management [18] and which consider the unique preferences and behaviors of each prosumer, offering personalized recommendations for optimizing energy usage and participation in LEMs.

There are several innovative technologies and platforms in the energy sector designed to support prosumers, such as (a) HEMSs like Nest (Google Nest), well-known for its smart thermostats which learn schedules and preferences to optimize heating and cooling for energy efficiency [19], and Tesla Energy, which offers solar panels, solar roof and battery systems, integrating with a mobile app for energy monitoring and management [20]; (b) DR and energy optimization services, like OhmConnect, which rewards users for saving energy during peak hours, integrating with smart home devices to automate energy savings, and AutoGrid, which uses big data analytics and AI to offer DR, distributed energy resource management and energy storage optimization; (c) P2P energy trading platforms, like LO3 Energy (Exergy), a blockchain platform enabling LEM for P2P energy trading, and Power Ledger, which utilizes blockchain technology to facilitate energy trading, allowing consumers to buy and sell renewable energy directly [21]; (d) predictive analytics and AI for energy management, like Bidgely, which utilizes AI and machine learning (ML) to disaggregate energy data from smart meters, providing personalized energy insights and recommendations [22,23], and Tibber, a digital electricity supplier that uses AI to optimize electricity consumption for its customers, offering dynamic pricing based on real-time market conditions; (e) platforms integrating LLMs, which are a cutting-edge area of development and not yet sufficiently investigated. They have not been applied to prosumers’ energy systems and thus we identified a research gap.

While specific platforms and solutions may vary, the integration of Internet of Things (IoT) devices, smart meters, blockchain platforms, and advanced analytics forms the backbone of modern recommendation systems for energy prosumers. They have the potential to transform energy markets and consumer behavior in several impactful ways: (a) Empowerment and autonomy for consumers. By providing personalized insights and actionable recommendations, these systems empower consumers, turning passive users into active participants in the energy market [24]. (b) Enhanced energy efficiency. Through optimized scheduling and operation of appliances, heating, cooling and lighting based on real-time data and predictive analytics, these systems significantly reduce wasted energy [25]. (c) Support for RES integration. By efficiently managing the production and consumption of RESs, these systems facilitate a smoother integration of RES into the grid. (d) For prosumers, the ability to sell excess energy creates new economic opportunities. P2P trading platforms democratize energy markets, potentially leading to more competitive prices and innovative services [26].

In this paper, we propose a recommendation system that integrates heterogenous datasets and provides advice to prosumers to improve the consumption profile and trade on LEM. The novelty of our approach consists in building datasets and several scenarios that are handled using LLM technology, namely the OpenAI and Zero-Shot-GPT classifiers in order to extract meaningful recommendations at high frequency—5 min. These recommendations allow prosumers to adjust consumption (load) or trade on LEMs, by obtaining suggestions driven by data.

The current research is structured in several sections. In the next section, a brief literature review in this field is provided offering a comparison of the research’s main focus, outcomes, related fields, involved technologies and whether they include large language models (LLMs) or AI. Section 3 is focused on the proposed methodology to handle input datasets and obtain recommendations. In Section 4, two scenarios are shown, and their results are presented, whereas in Section 5, the insights of these analyses are summarized, and the main conclusions related to recommendation systems based on LLMs are drawn.

## 2. Literature Review

Recent years have witnessed a significant transformation in traditional power systems, primarily due to the extensive integration of RESs. This shift has notably included the emergence of residential consumers as prosumers, challenging the conventional operation of electricity markets. This evolution introduces both new challenges and opportunities, leading to the development of new business models (BMs). A key focus is the shift towards a prosumer-centric model, which encourages greater participation of small consumers in power systems. Ref. [9] explored the role of recent BMs in facilitating the growing influence of prosumers. It covered the definition of prosumers, their regulations, potential market designs in power systems, and the technologies enabling the rise of prosumer-driven markets. Furthermore, this research reviewed current and emerging BMs and discussed their future implications for modern power systems, along with recommendations to support BMs. It concluded that while innovative BMs are economically feasible, regulatory barriers may limit their global dissemination.

Furthermore, the energy transition’s momentum has necessitated utilities to adopt new strategies for managing local energy demand and supply, owing to the increasing prevalence of prosumers. Addressing this requires an enhanced understanding and management of local energy consumption and production patterns. Small municipal utilities face particular challenges due to their lack of access to advanced modelling and forecasting tools. In this context, ref. [27] proposed a user-centered visual analytics approach for developing a tool that facilitates interactive and explainable day-ahead forecasting and analysis in prosumer environments. This research included the use of behavioral analysis to examine the connection between consumption patterns and prosumer interaction with energy tools. By employing explainable machine learning (ML) methods like kNN and decision trees alongside interactive visualization, utility analysts could better understand consumption influencers and make more accurate demand forecasts under uncertain conditions.

Transactive energy management (TEM) introduced a pioneering P2P energy trading concept, aiming for enhanced sustainable energy use by involving local prosumers as key participants in the energy ecosystem [28]. Thus, addressing prosumer concerns is vital for adopting the P2P trading model, where regulatory and policy considerations play a significant role. This research simplified transactive energy to identify its core components and reviewed each to highlight their importance and efficiency. It further discussed enabling technologies for TEM systems and concluded with policy recommendations to accelerate adoption. Additionally, the incorporation of regulatory, security concerns and the use of non-fungible tokens (NFTs) and techniques like ML and IoT were proposed to optimize the TEM process by addressing technological constraints.

The complexity of multi-agent systems (MASs), along with developments in AI and LLMs, has exposed significant gaps in understanding agent behaviors and interactions in dynamic settings. However, traditional game theory’s effectiveness is limited by its static and homogeneous assumptions. To address this, ref. [29] introduced an extended coevolutionary theory (ECT) that incorporates coevolutionary dynamics, adaptive learning, and LLM-based strategic insights to analyze heterogeneous agent interactions in MASs. This framework transcended game theory by considering diverse interactions, risk preferences and learning abilities among agents. The researchers demonstrated the ECT effectiveness through a simulation that explored cooperation and defection patterns, indicating its potential to foster cooperative behaviors and system robustness. This research highlighted the role of LLMs in enhancing cooperation and strategic resilience in MASs, offering insights into strategic decision-making, adaptive learning and LLM-guided management in complex systems.

Android robots equipped with dialogue systems were further expected to offer advanced conversational capabilities, reliability, and hospitality, mimicking human interactions [30]. However, dialogue systems based solely on LLMs may produce irrelevant or contradictory responses. The researchers proposed a scenario-based system that breaks down tasks into sub-tasks like summarization and response generation, utilizing LLMs more effectively to address these issues. This system, tested in a tourist-spot recommendation competition, showcased superior performance over rule-based systems, highlighting both the potential and challenges of integrating LLMs in android dialogue systems, such as computational delays and coordination with robot movements.

ChatGPT version 3.5 and similar LLM-based conversational agents have revolutionized research with their human-like performance. However, their predominantly reactive nature limits their ability to fully understand users and the context, missing out on proactive engagement opportunities like initiating conversations or offering context-aware recommendations. In this context, researchers explored methods to endow conversational agents with proactive abilities, covering system design, recent LLM advancements, and conversation management strategies [31]. Through interactive exercises, it aimed to enhance agents’ proactive interaction capabilities, thereby improving user engagement and safety in conversational applications.

Another research investigated how individuals react to recommendation options from ChatGPT across five studies [32]. Unlike prior studies on choice overload, findings revealed a positive response to a large array of options, showing varied consumer perceptions towards AI-generated recommendations. Further studies highlighted the influence of the recommendation source on consumer reactions and reveal a preference for ChatGPT, especially with numerous options. These insights were important for the design of recommendation systems and understanding user preferences for AI-generated recommendations.

Agenda 2030’s Sustainable Development Goals (SDGs) 9 and 11 underscored tourism’s pivotal role in addressing global challenges, with ICT transformations propelling e-tourism’s global evolution [33]. This research delved into contextual suggestion and recommendation systems within e-tourism, spotlighting approaches and their associated challenges through a systematic literature review of articles published between 2012 and 2020 across major repositories. The review followed a structured protocol, culminating in a taxonomy analysis that categorized the literature into review articles, models/frameworks, and applications. The analysis critically evaluated the limitations of current methods, predominantly content-based and collaborative filtering, alongside preference-based ranking and language modeling. Evaluation metrics and test collections for these systems were discussed, highlighting their relevance in achieving SDGs by integrating real-time data and web-based services for sustainable urban planning and development in the tourism sector. These recommendation and guidance systems based on LLMs and AI have been more extensively investigated for medicine [34,35,36], robotics [37], hospitality and tourism management [38], policy [39], education [40,41], etc.

As for the decision-making models, integrating expert judgments expressed in natural language via sentiment analysis may enrich decision-making processes. The sentiment analysis in recommender systems with multi-person, multi-criteria decision-making methods leveraged written expert reviews and ratings to inform decisions, addressing the challenge of information overload through intelligent recommender systems [42]. These systems, traditionally based on single-grading algorithms, were enhanced by multi-criteria systems that evaluate various product aspects. This research introduced a model that combines deep learning with multi-criteria decision-making, showcasing its effectiveness in providing accurate recommendations with a sentiment analysis accuracy of 98%. The system’s performance, evaluated through precision, recall and F1 scores, marked a significant improvement over previous models, demonstrating deep learning’s potential in refining recommender systems’ predictive capabilities and user satisfaction. In Table 1, we provide a summary of the information from the analyzed references.

This table encapsulates each research’s main focus, outcomes, related fields, involved technologies and whether they include large language models (LLMs) or AI.

## 3. Methodology

The input data may consist of the following variables at 15-min or even 5-min resolution: weather forecast, RES system output, schedule of the appliances, status: deficit/surplus, quantity of deficit/surplus, predicted prices on local energy market (LEM).

The prosumer may query his home assistant and obtain the updated information related to the weather forecast and RES system forecast in a speech format. This is actually a text format conveyed into speech by a home assistant. The entire input at a given moment becomes input for a classification problem that provides a recommendation out of a list of recommendations. Even if the input is not labeled, the indication is given to the prosumer considering a candidate list of recommendations. The potential recommendation output could be to adjust local consumption—increase or decrease, to trade on LEM—sell or buy.

The prosumer’s objective function is to minimize the electricity cost, that is, the product between consumption level (quantity) and price. The price could be the price from the LEM or the time-of-use price from the grid. Several constraints can be formulated. The indoors constraints when sensing the presence of humans could be related to the temperature = 21–23 °C during winter season; 23–24 °C during summer season.

To formulate the given scenario as a mathematical optimization problem, we will define the input parameters, decision variables, the objective function and the constraints of the problem.

Input parameters:

$W_{t}$—Weather forecast at time *t* in text format;
${RES}_{t}$—Output of RES system at time *t*.
$S_{t}$—Schedule of the appliances at time *t*.
$D_{t}$—Energy status at time *t* (deficit or surplus).
$Q_{D_{t}}$—Quantity of deficit or surplus at time *t*.
$P_{LEM_{t}}$—Predicted price on LEM at time *t*.

The input parameters may contain performance metrics such as self-sustainability index (SSI) for prosumers that represents an index for measuring the effectiveness with which individuals or households produce and use their energy, particularly from renewable sources such as photovoltaics (PV). The SSI indicates the proportion of a prosumer’s energy consumption that is met by their own renewable energy production. The SSI can be described as the percentage of the energy consumption that is both generated and used by the prosumer, showing how much of their energy needs are met independently. SSI thresholds (SST) are given in Equation (2).

(1) $$SSI\;\left(\%\right)=\frac{Selfgenerated\;energy\;consumed}{Energy\;consumed}\times 100$$

(2) $${SST}_{∆d}^{t}=\left\{\begin{array}{c} \mathrm{low},\;\mathrm{if}\;{SSI}_{∆d}^{t}<0.25\\ \mathrm{emerging},\;\mathrm{if}\;{SSI}_{∆d}^{t}\geq 0.25\;\mathrm{and}\;{SSI}_{∆d}^{t}<0.5\\ \mathrm{moderate},\;\mathrm{if}\;{SSI}_{∆d}^{t}\geq 0.5\;\mathrm{and}\;{SSI}_{∆d}^{t}<0.75\\ \mathrm{high},\;\mathrm{if}\;{SSI}_{∆d}^{t}\geq 0.75 \end{array}\right.$$

The self-sufficiency thresholds for the self-sustainability index (SSI) provide a structured approach to assess the sustainability levels of prosumers based on their energy generation systems. These thresholds classify prosumer sustainability into four distinct categories: (1) low sustainability (SSI < 25%) encompasses prosumers who generate a minor portion of their energy needs; (2) emerging sustainability (25% ≤ SSI < 50%) describes prosumers who are somewhat sustainable but still rely significantly on external energy sources; (3) moderate sustainability (50% ≤ SSI < 75%) applies to prosumers who cover a substantial portion of their energy needs through self-generation; (4) high sustainability (SSI ≥ 75%) is attributed to prosumers who meet most of their energy requirements from renewable sources.

The self-consumption index (SCI) serves as a measure for prosumers to gauge the proportion of the energy they generate that is directly consumed versus the amount fed back into the grid or left unused. This metric is particularly significant for those with renewable energy setups like PV systems. The SCI calculation is straightforward and involves determining the ratio of self-consumed energy against the total generated.

(3) $$SCI\;\left(\%\right)=\frac{Selfconsumed\;energy}{Generated\;energy}\times 100$$

(4) $${SCT}_{∆d}^{t}=\left\{\begin{array}{c} \mathrm{minimal},\;\mathrm{if}\;{SCI}_{∆d}^{t}<0.25\\ \mathrm{lower},\;\mathrm{if}\;{SCI}_{∆d}^{t}\geq 0.25\;\mathrm{and}\;{SCI}_{∆d}^{t}<0.5\\ \mathrm{moderate},\;\mathrm{if}\;{SCI}_{∆d}^{t}\geq 0.5\;\mathrm{and}\;{SCI}_{∆d}^{t}<0.75\\ \mathrm{high},\;\mathrm{if}\;{SCI}_{∆d}^{t}\geq 0.75 \end{array}\right.$$

Similar to SST, various threshold values of SCI (as in Equation (4)—SCT) have been established to classify self-consumption levels: (1) high self-consumption (SCI ≥ 75%) indicates that a significant majority of the energy produced is used directly by the prosumer, reflecting a high efficiency in power utilization; (2) moderate self-consumption (50% ≤ SCI < 75%) suggests that while a good portion of energy is utilized directly, there is still potential to enhance efficiency; (3) lower self-consumption (25% ≤ SCI < 50%) reflects that a substantial amount of generated energy is not being directly consumed, highlighting potential inefficiencies; (4) minimal self-consumption (SCI < 25%) shows that most of the generated energy is not being used by the prosumer, suggesting a significant misalignment between generation and consumption patterns. These structured recommendations provide a blueprint for prosumers at different levels of self-consumption to optimize their energy systems and enhance sustainability.

Grid dependence index (GDI) is a metric designed to quantify how much a prosumer relies on the grid to meet their energy needs. This index is calculated using a specific formula that contrasts the amount of energy consumed from the grid with the total energy consumption of the prosumer. The purpose of the GDI is to highlight how dependent a prosumer is on the power grid.

(5) $$GDI\;\left(\%\right)=\frac{Energy\;purchased\;from\;the\;grid}{Energy\;consumed}\times 100$$

(6) ESI=Energy cost from the Grid−Selfgenerated energy cost

The economic savings index (ESI) quantifies the cost savings realized by a prosumer from using their own generated energy versus the alternative of purchasing the equivalent amount of energy from the grid. It effectively measures the economic impact of self-sufficiency in energy production.

Decision variables:

$C_{t}$—Consumption adjustment at time *t* (Increase or Decrease).
$T_{LEM_{t}}$—Trading action on LEM at time *t* (Sell or Buy).

Objective Function:

To minimize the total cost over the considered time horizon *T*, the objective function can be expressed as:

(7) $$Minimize\sum_{t=1}^{T}Q_{C_{t}}\times P_{LEM_{t}}$$

where $Q_{C_{t}}$ is the adjusted quantity of consumption or trading (selling/buying) at time *t*.

Constraints:

1. Energy balance constraint ensures that energy consumption and production are balanced after any adjustments and trading actions.
  (8) $$Q_{{RES}_{t}}+Q_{T_{LEM_{t}}}+Q_{C_{t}}=Q_{D_{t}}\forall t$$
  where $Q_{{RES}_{t}}$ is the energy produced from RESs, $Q_{T_{LEM_{t}}}$ is the quantity traded on the LEM (positive for buying, negative for selling).
2. Indoor temperature constraints maintain indoor temperature within specified ranges according to the season, when human presence is detected.
  - Winter: $21\;{}^{\circ}C\leq T_{indoor}\leq 23\;{}^{\circ}C$
  - Summer: $23\;{}^{\circ}C\leq T_{indoor}\leq 24\;{}^{\circ}C$

These constraints imply the adjustment of energy consumption for heating/cooling as part of $C_{t}$.

Notes:

- *T* is the set of discrete 15-min intervals considered.
- The decision variables $C_{t}$ and $T_{LEM_{t}}$ must be chosen such that they satisfy all constraints for each time interval *t*.

Multiple datasets are usually inserted into the model. The weather forecast is usually a text or json format data that can be added to the rest of the variables to create a list of string elements that forms the input of a classification problem. To process the text data, ChatGPT showcases an impressive feature: the ability to categorize text without undergoing specific training by leveraging descriptive labels for effective classification. ZeroShotGPTClassifier is a component of skllm, designed to streamline the creation of text classification models. It does not require text pre-processing pipelines.

This tool parallels the simplicity and functionality of classifiers found in the scikit-learn library. Essentially, the ZeroShotGPTClassifier taps into ChatGPT’s inherent skill to interpret and classify text using labels, offering a straightforward approach to text classification devoid of the usual training hurdles.

The two datasets for the classification problem can be defined as

(9) $$X=[“{text}_{1}”,“{text}_{2}”,\ldots \;“{text}_{n}”]\;y=[“{label}_{1}”,“{label}_{2}”,\ldots \;“{label}_{n}”]$$

The text input may include weather forecast and RES system output, whereas the label can be to adjust consumption and trade on the LEM.

The *X* and *y* are split into train and test, and an OpenAI model is applied to train the classifier. The prediction is performed on test data, and the results are paired with their input for analysis.

The interesting part lies in the fact that there is no need for prelabeled data to train the model but a set of possible labels to begin with. Thus, the labels list is passed only for prediction. This method unveils opportunities for training models in scenarios where access to pre-labeled datasets might not be available.

**Figure 1:**
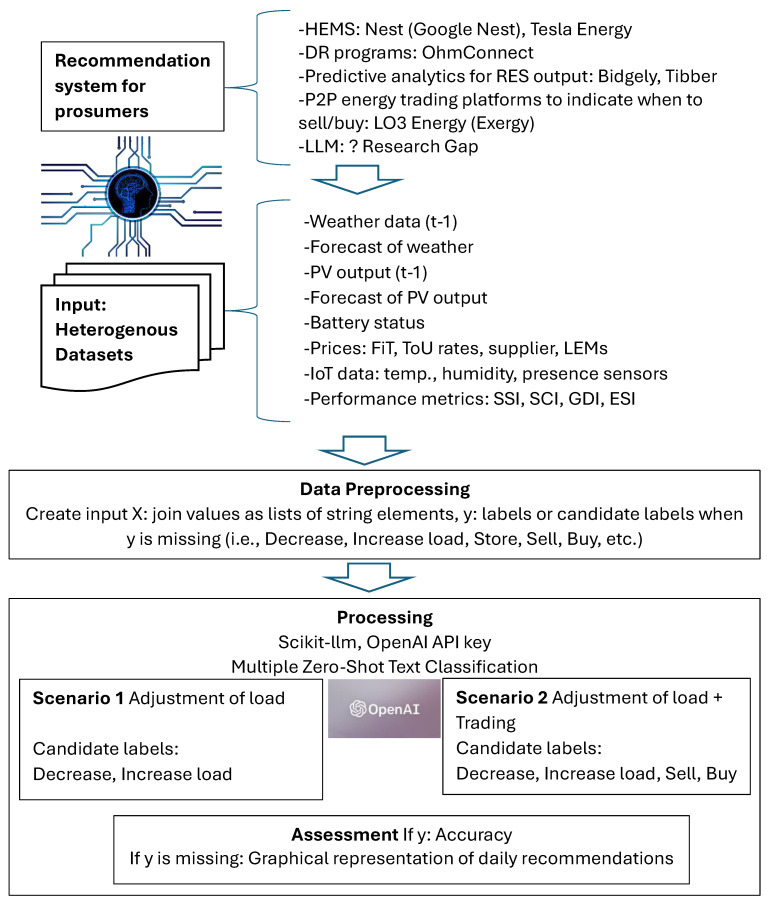
Methodology processing flow.

The proposed methodology flow is presented in Figure 1.

## 4. Results

For simulations, a prosumer with a PV system (of 5.9 kW) output located in rural area is used, as well as weather forecast and the PV system output forecast. Data readings from an inverter were recorded at 5-min intervals, whereas weather and PV system forecast are obtained at 1-h intervals. The reading date was used to merge the datasets and the missing data due to the different resolutions were filled in using bfill for weather forecasting and interpolate methods for PV system forecasting. While our primary objective did not focus to explore diverse data preprocessing methods, we did examine various approaches for addressing missing values. Among these methods, the manuscript provides the most effective solutions we identified. It is worth noting that while interpolation, like any method for handling missing data, may introduce inaccuracies, it is preferable to approximate PV output through interpolation rather than leaving these values as null.

To ensure that the data fed into the LLMs for the zero-shot classifier-based recommender system is thoroughly preprocessed and validated, a comprehensive approach needs to be adopted. This process involves multiple stages of data handling, from initial collection to final validation, ensuring the accuracy and reliability of the inputs.

The first step is data collection, which involves gathering data from diverse sources, including PV system outputs, weather forecasts, energy consumption patterns, grid prices, and IoT sensor readings like temperature and humidity. It is significant to ensure that data is collected at consistent intervals, such as 5-min inverter readings and 1-h weather forecasts, to maintain temporal coherence. Next, data cleaning is performed to remove any duplicates or erroneous entries. Missing data points are handled using backward fill for weather forecast gaps and interpolation techniques for PV system output gaps. Data formats are normalized to ensure consistency across different data sources.

Data transformation follows, converting raw data into structured formats suitable for LLM processing. Data is aggregated into meaningful time intervals (such as daily or hourly, as required). Data validation is a critical step where data is cross-checked against known benchmarks or historical data to verify accuracy. Automated validation scripts are implemented to flag any anomalies or outliers. Statistical methods are used to ensure the integrity of data distributions, such as mean and variance checks.

The following attributes (as in Table 2) are considered to classify the output of the recommendation system using multilabel zero-shot text classification.

ChatGPT demonstrates a notable ability: it can categorize text without dedicated training, using merely descriptive labels to efficiently perform this task. Introducing the ZeroShotGPTClassifier from Scikit-LLM, this tool allows users to easily create a text classification model comparable to other classifiers in the scikit-learn library. Essentially, the ZeroShotGPTClassifier leverages ChatGPT’s distinctive capability to comprehend and classify text based on labels, streamlining the text classification process and eliminating the need for conventional training. The most important library is scikit-llm that has to be imported, but depending on the environment, other libraries can be required (such as cython, watermark, skllm.config).

The compelling part is that there is no need for prelabeled data to train the model. Only a list of potential candidate labels is required. This method allows for the training of models even in scenarios where one lacks access to pre-existing labeled datasets.

The key difference between zero-shot and multilabel zero-shot classification is simply the instantiation of the MultiLabelZeroShotGPTClassifier class. For multilabel zero-shot classification, one defines the maximum number of labels to assign to each sample, such as specifying max_labels = 3. This parameter gives the ability to control the number of labels the model can assign to a text sample during the classification process.

**Figure 2:**
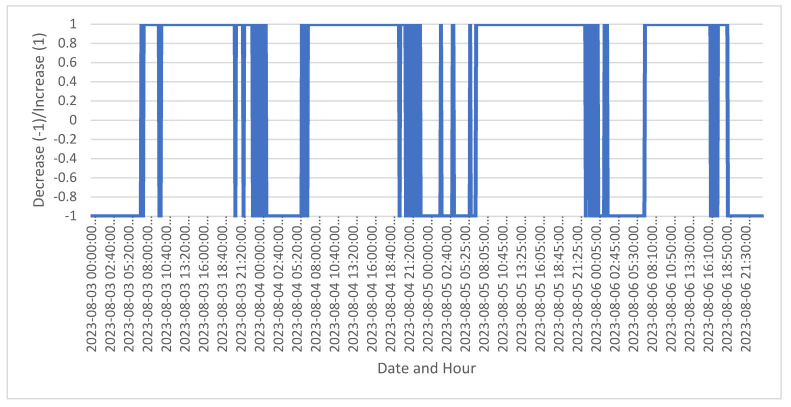
Output of 5-min recommendations from 3 to 6 of August 2023 using 2 potential labels.

For simulation, a free instance of Google Collaboratory was used. In the first scenario, when the candidate labels are candidate_labels = [“Increase”, “Decrease”], the following output of 5-min recommendations from 3 to 6 August 2023 with the two potential labels are obtained (as in Figure 2). The input data is extracted from a larger dataset from 1 of August until 15 of August 2023, and consists of 4301 rows and 11 columns. The graphical representation is significant as y test is missing, and therefore accuracy cannot be calculated.

This visualization shows the distribution and frequency of the recommendations “Increase” and “Decrease” load across the specified timeframe. Each row represents a different 5-min interval with a recommendation derived from the ZeroShotGPTClassifier. The effectiveness of the ZeroShotGPTClassifier in this scenario largely depends on the capability of the underlying model (e.g., ChatGPT) to interpret the context accurately.

The input data format is structured in a way that is compatible with the classifier, typically requiring preprocessing to extract relevant textual information from each time interval. Each input data was joined into a list of string elements. This first scenario effectively showcases how advanced natural language processing tools can be leveraged for dynamic and context-sensitive decision-making processes in a highly granular manner.

Figure 2 shows 5-min interval recommendations from 3 August to 6 August 2023, with the potential labels “Increase” (1) and “Decrease” (−1). An interpretation of the chart is provided: the Y axis represents recommendations as binary outcomes. The value 1 indicates a recommendation to “Increase” load and −1 indicates a recommendation to “Decrease” load, whereas the X axis shows dates and times, marked at 5-min intervals over four days, from 3 August to 6 August 2023. It shows alternating periods where the recommendation switches between “Increase” and “Decrease” load. This suggests a dynamic situation where conditions or factors evaluated at these intervals are frequently changing. Some hours (like night and day hours) show a consistent recommendation (either to increase or decrease), which suggest more stable or predictable conditions during these periods. Other periods, notably around midday across the days, show more frequent switches between “Increase” and “Decrease” load. This reflects more volatile or variable weather conditions needing quick responses. Each day might have slightly different patterns, which are influenced by different external factors not shown on the chart but affecting the recommendations. This chart provides a clear, quick reference for actions recommended at regular intervals, reflecting an automated, data-driven decision process. In Table 3, sample data for the first scenario is provided.

For the second scenario, we added the prices for selling and buying energy from local market or supplier (as in Table 4). Price_buy_from_grid is a time-of-use tariff that is designed to encourage load at the off-peak hours. The candidate labels are candidate_labels = [“Increase”, “Decrease”, “Sell”, “Buy”].

**Figure 3:**
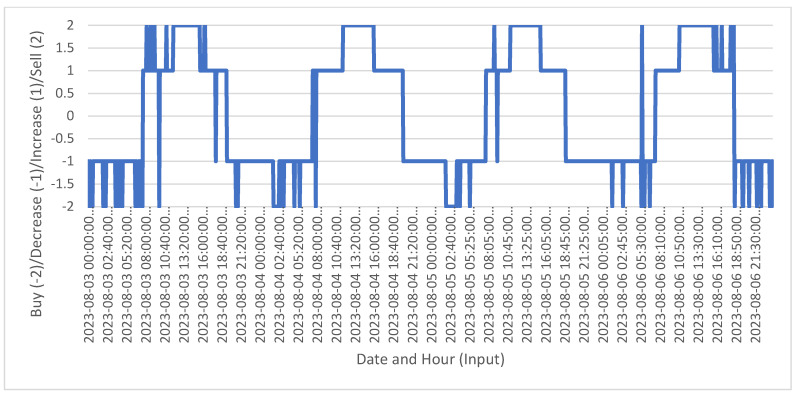
Output of 5-min recommendations from 3 to 6 of August 2023 using 4 potential labels.

The results of the simulation for the second scenario, which took place for the same interval, are presented in Figure 3.

Figure 3 shows recommendations over a range of values from 3 August to 6 August 2023, labeled on the Y axis as Buy (−2)/Decrease (−1)/Increase (1)/Sell (2). An interpretation is provided: the Y axis extends beyond binary outcomes to include values of 2, 1, −1 and −2. The values 1 and −1 still correspond to “Increase” and “Decrease” load recommendations, while 2 and −2 correspond to stronger recommendations like “Buy” and “Sell”, respectively, whereas the X axis displays dates and times across the specified range, marked at 5-min intervals. Positive values (2 and 1) indicate varying degrees of bullish recommendations. A value of 2 (Sell) suggests a strong action, while a value of 1 (Increase) suggests a moderate action. Negative values (−1 and −2) indicate varying degrees of bearish recommendations. A value of −1 (Decrease) suggests a moderate action, while a value of −2 (Buy) suggests a strong action. The chart shows significant fluctuations between the values, suggesting rapidly changing conditions or responses to new data. There are periods where the recommendations stay constant for a series of intervals, indicating stable conditions or sustained decisions based on the underlying data. Some intervals show sharp transitions from strong to moderate recommendations or vice versa, which indicate volatile conditions or moments of significant change in metric or data the model is assessing. Sample data for the second scenario is provided in Table 5.

The recommendations depicted in Figure 3 are based on several sets of input data attributes that influence the decision-making process for actions such as “Buy”, “Sell”, “Increase”, and “Decrease”. These attributes potentially impact the recommendations: (1) READING_DATE (Date and Hour) serves as the temporal marker for each data point, aligning recommendations with specific times and showing patterns over daily cycles; (2) OPEN_WEATHER (Weather forecast) directly impacts PV system output predictions and can influence decisions about power management based on anticipated solar generation capacity; (3) POWER_FORECAST (PV system output forecast) is a critical input for planning whether to store energy, sell surplus or manage deficits, impacting “Increase” and “Sell” decisions; (4) POWER_LOAD (consumption power) determines how much power is needed at any given time, influencing “Decrease” or “Increase” in load management; (5) POWER_GEN (generated power) is the actual power generation data influences real-time decisions on whether there is a surplus to sell or a need to draw from other sources; (6) POWER_BAT (power extracted from battery) indicates decisions on whether to draw power from the battery or to charge it depend on other power availability and demands; (7) POWER_GRID (power extracted from grid) indicates the usage of grid power indicates whether to buy additional power or manage with generated or stored power; (8) SD_CAPACITY (battery capacity state of charge) affects decisions on battery charging or discharging strategies; (9) LOAD_PERCENT (percentage of the load from rated power of the PV system) indicates how heavily the system is loaded compared to its capacity, influencing load management strategies; (10) VPV (voltage of the PV system) and (11) IPV (current of the PV system) inform about the operational status and efficiency of the PV system, affecting decisions related to system load and generation management. Additional attributes, such as (12) Price_sell_to_grid also known as feed-in-tariff, (13) Price_buy_from_grid usually tariff rates that takes into account the consumption moment, (14) Price_sell_to_LEM and (15) Price_buy_from_LEM are economic factors and play a critical role, as the decision to buy or sell power (either to/from the grid or a LEM) is influenced by these prices.

The cost-effectiveness of each action (buying or selling) dictates whether it is more advantageous to generate, store or purchase power. Each recommendation, whether it is “Decrease” or “Increase” in load, or “Sell” and “Buy” in terms of energy transactions, is derived from analyzing these diverse data inputs. The complexity and variability of these attributes underpins the advanced analytics required to optimize energy management in real time.

Creating a recommendation system for prosumers in local electricity markets using a zero-shot classifier is an innovative approach, particularly because it leverages the ability of zero-shot learning to handle data categories (like specific market conditions or consumer behaviors) that were not seen during training. This kind of system can be compared to several existing types of recommendation systems, each having distinct mechanisms and applications. An overview of different recommendation systems and how they might relate to or differ from zero-shot classifier-based systems is provided in Table 6.

Collaborative filtering is one of the most common techniques used in recommendation systems and relies on gathering and analyzing data on user behavior, activities, or preferences to predict what a user will like, based on similarity to other users. This system does not use zero-shot capabilities but is effective in environments with rich user interaction data. These systems provide helpful output, but they require a large amount of user data and struggle with new items (also known as the cold start problem).

Content-based filtering is another technique that recommends items similar to those a particular user has liked in the past, based solely on the content (features) of the items rather than user interactions. It uses item features to make predictions (conventional classifications), which could be somewhat similar to using a zero-shot classifier in that it can handle new items more effectively than collaborative filtering. These systems can handle new items better since it does not rely on user-user similarities, but they provide less personalization compared to collaborative filtering, as it does not consider user interaction patterns.

Hybrid systems combine collaborative and content-based filtering to improve recommendation quality and overcome the limitations of both systems. These could be analogous to using zero-shot classification alongside other machine learning models to provide energy usage recommendations. These systems balance the advantages of both content-based and collaborative filtering. However, they require complexity in implementation and integration of different recommendation logic.

Knowledge-based systems recommend products based on specific domain knowledge about how certain item features meet user needs and preferences. They often include rule-based systems that do not require user data history but rely on an inference from known characteristics, which is somewhat similar to zero-shot learning in that it can make inferences without direct previous examples. These systems are effective where user preferences are known and can handle complex user requirements. However, they require extensive domain knowledge and manual rule setup.

Utility-based recommenders make suggestions based on a computation of the utility of each item for a user. This could involve complex calculations where different attributes of items are weighed against user preferences. Zero-shot classifiers could potentially be used to estimate such utilities based on descriptions or attributes without prior examples. These systems are highly customizable and can be adapted to different user specifications. They require a detailed model of user preferences and item utilities.

To compare the results of our approach and a classic recommendation system, we calculate the performance metrics such as self-sustainability index (SSI), self-consumption index (SCI), grid dependence index (GDI), and economic savings index (ESI), described in equations (1), (3), (5) and (6). These metrics are relevant for prosumers, and they indicate the efficiency of a recommendation system. Thus, we compare the performance metrics obtained with both systems: a zero-shot text classifier-based recommender system versus a content-based filtering system for a prosumer over a month (as in Table 7). They reflect the superior performance of the zero-shot text classification system in optimizing energy usage and economic benefits. Over a month, the zero-shot classifier-based recommender system demonstrates superior performance in all metrics compared to the content-based filtering system. It achieves higher self-sustainability and self-consumption rates, lower grid dependence, and greater economic savings. This indicates that the zero-shot classifier-based approach is more effective in optimizing energy usage and providing economic benefits to prosumers.

These estimates highlight the potential improvements in energy management and economic efficiency that can be achieved with a more advanced recommendation system leveraging zero-shot classification.

## 5. Conclusions

This paper outlines the deployment of modern technologies such as home assistant devices and recommendation systems in energy management, specifically tailored for homeowners who also act as prosumers. These systems utilize detailed data collected at short intervals, including weather forecasts, energy production and consumption patterns to provide real-time energy management solutions. The goal is to optimize energy consumption, reduce costs and maintain comfort by dynamically adjusting energy usage or participating in energy trading through LEMs.

The proposed methodology outlines a recommendation system for prosumers. The system uses a variety of data inputs and processes to optimize both energy usage and trading decisions. Alternative systems are diverse, including home energy management systems like Nest or Tesla Energy, Demand Response programs such as OhmConnect and predictive analytics for renewable energy sources output from platforms like Bigdely and Tibber. It may also include data from P2P energy trading platforms, weather and PV output forecasts, battery status and pricing information including feed-in tariffs, time-of-use rates and LEM prices. Additional inputs may include Internet of Things data such as temperature, humidity and sensor data along with performance metrics like the self-sustainability index, self-consumption index, grid dependence index, economic savings index.

In the data preprocessing stage, several inputs are prepared and combined into a usable format. For instance, values are joined as lists of strings, or labels are processed for candidate actions when labels are missing. The processing stage involves using tools like Scikit-llm and the OpenAI API for natural language or other data processing tasks. It also includes multiple zero-shot text classification to determine actions. The proposed recommendation system supports multiple operational scenarios. The first scenario involves adjusting the load dynamically, increasing or decreasing energy load based on real-time data and forecasts. The second scenario adds the capability to make real-time decisions about energy trading, such as selling excess energy or buying additional energy as needed. The final step in the system involves assessing the accuracy of the outputs: y predicted is compared with y test. If key data (y) is missing, the system provides a graphical representation of daily recommendations.

For simulations, a prosumer with a 5.9 kW PV system located in a rural area utilizes a sophisticated recommendation system to optimize energy management based on various data inputs and machine learning and LLM techniques. The system processes data from multiple sources including inverter readings, weather forecasts and PV output forecasts to inform real-time decision-making. The data collected include inverter readings at 5-min intervals and forecasts at 1-h intervals. Due to the varying resolutions, data merging is conducted based on reading dates, with missing data points filled using backward fill for weather forecasts and interpolation for PV system forecasts. Attributes such as date, weather forecast, power forecasts, consumption, generated power, battery status, grid dependency and more are considered to classify the output of the system using multilabel zero-shot text classification. This classification approach leverages the ZeroShotGPTClassifier from Scikit-llm, allowing the system to categorize data without pre-existing labeled datasets. The classifier uses descriptive candidate labels to understand and assign categories, making it particularly useful in dynamic and data-rich environments.

The data spanning from 1 to 15 August 2023, with 4301 data points was processed to predict actions such as “Increase” or “Decrease” energy load. A graphical representation of these recommendations over several days illustrates the decision-making process of the system. Recommendations are made every five minutes, reflecting the rapid analysis and response capabilities of the system. Further expanding the simulation to include energy trading, additional labels such as “Buy” and “Sell” are introduced, taking into account the prices for energy transactions. This allows the system not only to manage energy efficiently but also to engage in energy trading based on real-time market conditions. The effectiveness of the ZeroShotGPTClassifier and the entire system is depicted through detailed graphs showing recommendations over time. These visualizations demonstrate how the system adapts to changing conditions, recommending different actions based on a complex set of input attributes. These attributes include operational data from the PV system, the state of battery charge and economic factors such as local energy prices.

This research highlights the integration of large language models (LLMs) and other algorithms in developing novel recommendation systems without using complex pipelines for data pre-processing. These systems analyze various data points to offer personalized advice, aiming to efficiently manage energy production and consumption, engage in profitable energy trading and utilize incentives such as demand response programs. Predictive analytics for renewable energy production and peer-to-peer energy trading platforms also play crucial roles in enhancing the financial and environmental efficiency of energy systems.

Furthermore, the use of artificial intelligence, including LLMs, to analyze and process vast datasets without the need for pre-labeled data represents a significant advancement in recommendation systems. This approach allows for the development of flexible, adaptive systems that can handle complex, dynamic data sets in real-time, providing tailored recommendations to prosumers. These novel systems support the integration of renewable energy sources and potentially transform energy markets through innovative services.

Future works based on the deployment of modern technologies and recommendation systems in energy management for prosumers can explore several promising areas. Enhancing data integration and processing methods is crucial. Our future research will investigate more efficient algorithms and frameworks for seamlessly integrating and processing diverse data sources, including IoT sensors, weather forecasts, PV output and market prices that would enhance the decision-making capabilities of recommendation systems. Moreover, developing comprehensive performance metrics and benchmarking tools to evaluate the effectiveness of LLM-based recommendation systems will be necessary.

There are several limitations that could be enhanced in the future. The lack of interpretability and transparency of recommender systems may raise concerns regarding user trust and adoption, especially when deploying complex systems like LLMs and zero-shot classifiers. Addressing these concerns is important for ensuring users understand and have confidence in the recommendations provided by the system. To increase interpretability and transparency, explanations could be generated alongside each recommendation provided by the zero-shot classifier. These explanations should highlight the key factors influencing the recommendation, such as current energy consumption patterns, weather conditions or predicted energy production.

Moreover, a feature importance analysis could be conducted using techniques like SHAP (Shapley additive explanations) or LIME (local interpretable model-agnostic explanations). These methods decompose the model’s predictions into contributions from each feature, providing insights into how different inputs influence recommendations. A feedback mechanism will allow users to provide input on the recommendations and explanations provided by the system. User feedback helps identify areas for improvement and ensure that recommendations align with user expectations. By addressing these points, we plan to enhance the interpretability and transparency of the zero-shot classifier-based recommender system.

## Figures and Tables

**Table 1:** Comparative analysis of the investigated references.

Ref.	Objective	Brief Results	Field	Technologies	Includes LLM/AI
[9]	Discuss recent business models as enablers of increasing prosumers’ role in power systems.	Comprehensive review of existing and innovative business models, discussion on future roles, and set of recommendations.	Power Systems	Enabling technologies for new prosumer-driven markets.	No
[27]	Develop a tool for day-ahead forecasting and analysis of energy demand in local prosumer environments using a visual analytics approach.	Suggestion of using explainable ML methods and interactive visualization for understanding consumption patterns.	Energy Management	Explainable machine learning (kNN, decision trees), interactive visualization.	Yes
[28]	Explain transactive energy and examine enabling technologies for P2P energy trading involving prosumers.	Discussion on key components of transactive energy systems and policy recommendations for adoption.	Energy Trading	Machine Learning, IoT, NFTs.	Yes
[29]	Propose an Extended Coevolutionary Theory for modeling strategic interactions in Multi-Agent Systems (MASs) with AI and LLMs.	Development of a simulation environment and visualization of cooperation and defection patterns in MASs.	Multi-Agent Systems	AI, Large Language Models.	Yes
[30]	Develop a hospitable dialogue system for android robots using LLMs in a fine-grained manner.	Achieved high placement in Dialogue Robot Competition 2022, identified challenges with LLMs in android systems.	Robotics, Dialogue Systems	Large Language Models.	Yes
[31]	Review methods for equipping conversational agents with proactive interaction abilities.	Discussion on enhancing conversational agents’ proactiveness, including LLM-based advancements.	Conversational Agents	LLMs, Reinforcement Learning with Human Feedback (RLHF).	Yes
[32]	Examine consumer responses to recommendation options generated by ChatGPT.	Found preferences for AI-generated recommendations and distinct consumer reactions.	Consumer Behavior	AI-powered language model (ChatGPT).	Yes
[33]	Survey literature on contextual suggestion and recommendation systems in e-tourism.	Identified gaps and critical analysis of current approaches, discussion on implications for SDGs.	E-Tourism	Contextual suggestion systems, recommendation systems.	No
[42]	Present SAR-MCMD method for incorporating expert judgments into recommender systems through sentiment analysis.	Suggested system demonstrated high accuracy and performance metrics in case studies.	Recommender Systems	Deep learning, sentiment analysis.	Yes

**Table 2:** Attributes description of input data.

No.	Attribute	Description
1	READING_DATE	Date and Hour
2	OPEN_WEATHER	Weather forecast
3	POWER_FORECAST	PV system output forecast
4	POWER_LOAD	Consumption power
5	POWER_GEN	Generated power
6	POWER_BAT	Power extracted from Battery
7	POWER_GRID	Power extracted from Grid
8	SD_CAPACITY	Battery capacity State of Charge
9	LOAD_PERCENT	Percentage of the load from rated power of the PV system
10	VPV	Voltage of the PV system
11	IPV	Current of the PV system

**Table 3:** Sample data for the first scenario.

Input	Predicted_Labels
2023-08-01 00:05:00 {“dt”: “2023-07-31 23:00:00”, “pop”: 0.29, “uvi”: 0, “temp”: 22.69, “clouds”: 92, “weather”: [{“id”: 804, “icon”: “04n”, “main”: “Clouds”, “description”: “overcast clouds”}], “humidity”: 77, “pressure”: 1011, “wind_deg”: 71, “dew_point”: 18.45, “wind_gust”: 1.78, “feels_like”: 23.03, “visibility”: 10,000, “wind_speed”: 1.78} 3.5 108 0 109 0.0 87 2 0.0 0.0	[‘Decrease’, ‘’]
2023-08-01 00:10:00 {“dt”: “2023-07-31 23:00:00”, “pop”: 0.29, “uvi”: 0, “temp”: 22.69, “clouds”: 92, “weather”: [{“id”: 804, “icon”: “04n”, “main”: “Clouds”, “description”: “overcast clouds”}], “humidity”: 77, “pressure”: 1011, “wind_deg”: 71, “dew_point”: 18.45, “wind_gust”: 1.78, “feels_like”: 23.03, “visibility”: 10,000, “wind_speed”: 1.78} 3.4444444444444446 102 0 109 0.0 86 2 0.0 0.0	[‘Decrease’, ‘’]
2023-08-01 00:15:00 {“dt”: “2023-07-31 23:00:00”, “pop”: 0.29, “uvi”: 0, “temp”: 22.69, “clouds”: 92, “weather”: [{“id”: 804, “icon”: “04n”, “main”: “Clouds”, “description”: “overcast clouds”}], “humidity”: 77, “pressure”: 1011, “wind_deg”: 71, “dew_point”: 18.45, “wind_gust”: 1.78, “feels_like”: 23.03, “visibility”: 10,000, “wind_speed”: 1.78} 3.388888888888889 118 0 109 0.0 86 2 0.0 0.0	[‘Decrease’, ‘’]
2023-08-01 00:20:00 {“dt”: “2023-07-31 23:00:00”, “pop”: 0.29, “uvi”: 0, “temp”: 22.69, “clouds”: 92, “weather”: [{“id”: 804, “icon”: “04n”, “main”: “Clouds”, “description”: “overcast clouds”}], “humidity”: 77, “pressure”: 1011, “wind_deg”: 71, “dew_point”: 18.45, “wind_gust”: 1.78, “feels_like”: 23.03, “visibility”: 10,000, “wind_speed”: 1.78} 3.333333333333333 111 0 109 0.0 86 2 0.0 0.0	[‘Decrease’, ‘’]
2023-08-01 12:05:00 {“dt”: “2023-08-01 12:00:00”, “pop”: 0, “uvi”: 6.45, “temp”: 29.53, “clouds”: 1, “weather”: [{“id”: 800, “icon”: “01d”, “main”: “Clear”, “description”: “clear sky”}], “humidity”: 42, “pressure”: 1010, “wind_deg”: 249, “dew_point”: 15.27, “wind_gust”: 0.61, “feels_like”: 29.37, “visibility”: 10,000, “wind_speed”: 0.8} 4299.0 98 125 -19 0.0 98 2 321.5 2.5	[‘Increase’, ‘’]
2023-08-01 12:10:00 {“dt”: “2023-08-01 12:00:00”, “pop”: 0, “uvi”: 6.45, “temp”: 29.53, “clouds”: 1, “weather”: [{“id”: 800, “icon”: “01d”, “main”: “Clear”, “description”: “clear sky”}], “humidity”: 42, “pressure”: 1010, “wind_deg”: 249, “dew_point”: 15.27, “wind_gust”: 0.61, “feels_like”: 29.37, “visibility”: 10,000, “wind_speed”: 0.8} 4292.0 107 117 -19 0.0 98 2 320.4 2.3	[‘Increase’, ‘’]
2023-08-01 12:15:00 {“dt”: “2023-08-01 12:00:00”, “pop”: 0, “uvi”: 6.45, “temp”: 29.53, “clouds”: 1, “weather”: [{“id”: 800, “icon”: “01d”, “main”: “Clear”, “description”: “clear sky”}], “humidity”: 42, “pressure”: 1010, “wind_deg”: 249, “dew_point”: 15.27, “wind_gust”: 0.61, “feels_like”: 29.37, “visibility”: 10,000, “wind_speed”: 0.8} 4285.0 98 118 -19 0.0 98 2 320.7 2.3	[‘Increase’, ‘’]
2023-08-01 12:20:00 {“dt”: “2023-08-01 12:00:00”, “pop”: 0, “uvi”: 6.45, “temp”: 29.53, “clouds”: 1, “weather”: [{“id”: 800, “icon”: “01d”, “main”: “Clear”, “description”: “clear sky”}], “humidity”: 42, “pressure”: 1010, “wind_deg”: 249, “dew_point”: 15.27, “wind_gust”: 0.61, “feels_like”: 29.37, “visibility”: 10,000, “wind_speed”: 0.8} 4278.0 110 123 -19 0.0 97 2 319.1 2.4	[‘Increase’, ‘’]

**Table 4:** Additional attributes for the second scenario-sample data.

Additional Attributes	Price_Sell_to_Grid	Price_Buy_from_Grid	Price_Sell_to_LEM	Price_Buy_from_LEM
EuroCents	9	14	11	12.5

**Table 5:** Sample data for the second scenario.

Input	Predicted_ Labels
2023-08-03 00:05:00 {“dt”: “2023-08-03 00:00:00”, “pop”: 0, “uvi”: 0, “temp”: 22.85, “clouds”: 59, “weather”: [{“id”: 803, “icon”: “04n”, “main”: “Clouds”, “description”: “broken clouds”}], “humidity”: 74, “pressure”: 1009, “wind_deg”: 46, “dew_point”: 17.97, “wind_gust”: 3.43, “feels_like”: 23.12, “visibility”: 10,000, “wind_speed”: 2.42} 14.166666666666666 76 0 69 0.0 86 2 0.0 0.0 0.09 0.14 0.11 0.125	[‘Buy’, ‘’, ‘’]
2023-08-03 00:10:00 {“dt”: “2023-08-03 00:00:00”, “pop”: 0, “uvi”: 0, “temp”: 22.85, “clouds”: 59, “weather”: [{“id”: 803, “icon”: “04n”, “main”: “Clouds”, “description”: “broken clouds”}], “humidity”: 74, “pressure”: 1009, “wind_deg”: 46, “dew_point”: 17.97, “wind_gust”: 3.43, “feels_like”: 23.12, “visibility”: 10,000, “wind_speed”: 2.42} 13.333333333333334 78 0 74 0.0 86 2 0.0 0.0 0.09 0.14 0.11 0.125	[‘Buy’, ‘’, ‘’]
2023-08-03 00:15:00 {“dt”: “2023-08-03 00:00:00”, “pop”: 0, “uvi”: 0, “temp”: 22.85, “clouds”: 59, “weather”: [{“id”: 803, “icon”: “04n”, “main”: “Clouds”, “description”: “broken clouds”}], “humidity”: 74, “pressure”: 1009, “wind_deg”: 46, “dew_point”: 17.97, “wind_gust”: 3.43, “feels_like”: 23.12, “visibility”: 10,000, “wind_speed”: 2.42} 12.5 75 0 74 0.0 85 2 0.0 0.0 0.09 0.14 0.11 0.125	[‘Decrease’, ‘’, ‘’]
2023-08-03 00:20:00 {“dt”: “2023-08-03 00:00:00”, “pop”: 0, “uvi”: 0, “temp”: 22.85, “clouds”: 59, “weather”: [{“id”: 803, “icon”: “04n”, “main”: “Clouds”, “description”: “broken clouds”}], “humidity”: 74, “pressure”: 1009, “wind_deg”: 46, “dew_point”: 17.97, “wind_gust”: 3.43, “feels_like”: 23.12, “visibility”: 10,000, “wind_speed”: 2.42} 11.666666666666666 71 0 74 0.0 85 1 0.0 0.0 0.09 0.14 0.11 0.125	[‘Decrease’, ‘’, ‘’]
2023-08-03 12:05:00 {“dt”: “2023-08-03 12:00:00”, “pop”: 0, “uvi”: 7.53, “temp”: 33.42, “clouds”: 0, “weather”: [{“id”: 800, “icon”: “01d”, “main”: “Clear”, “description”: “clear sky”}], “humidity”: 35, “pressure”: 1010, “wind_deg”: 239, “dew_point”: 15.87, “wind_gust”: 3.01, “feels_like”: 33.4, “visibility”: 10,000, “wind_speed”: 2.04} 4138.25 96 132 -19 0.0 98 2 317.9 2.6 0.09 0.14 0.11 0.125	[‘Sell’, ‘’, ‘’]
2023-08-03 12:10:00 {“dt”: “2023-08-03 12:00:00”, “pop”: 0, “uvi”: 7.53, “temp”: 33.42, “clouds”: 0, “weather”: [{“id”: 800, “icon”: “01d”, “main”: “Clear”, “description”: “clear sky”}], “humidity”: 35, “pressure”: 1010, “wind_deg”: 239, “dew_point”: 15.87, “wind_gust”: 3.01, “feels_like”: 33.4, “visibility”: 10,000, “wind_speed”: 2.04} 4148.5 105 104 -19 0.0 98 2 317.7 2.0 0.09 0.14 0.11 0.125	[‘Sell’, ‘’, ‘’]
2023-08-03 12:15:00 {“dt”: “2023-08-03 12:00:00”, “pop”: 0, “uvi”: 7.53, “temp”: 33.42, “clouds”: 0, “weather”: [{“id”: 800, “icon”: “01d”, “main”: “Clear”, “description”: “clear sky”}], “humidity”: 35, “pressure”: 1010, “wind_deg”: 239, “dew_point”: 15.87, “wind_gust”: 3.01, “feels_like”: 33.4, “visibility”: 10,000, “wind_speed”: 2.04} 4158.75 89 117 -19 0.0 98 2 317.0 2.3 0.09 0.14 0.11 0.125	[‘Sell’, ‘’, ‘’]
2023-08-03 12:20:00 {“dt”: “2023-08-03 12:00:00”, “pop”: 0, “uvi”: 7.53, “temp”: 33.42, “clouds”: 0, “weather”: [{“id”: 800, “icon”: “01d”, “main”: “Clear”, “description”: “clear sky”}], “humidity”: 35, “pressure”: 1010, “wind_deg”: 239, “dew_point”: 15.87, “wind_gust”: 3.01, “feels_like”: 33.4, “visibility”: 10,000, “wind_speed”: 2.04} 4169.0 101 126 -19 0.0 97 2 316.9 2.5 0.09 0.14 0.11 0.125	[‘Sell’, ‘’, ‘’]

**Table 6:** Comparison of various recommendation systems.

System Type	Requirements	Pros	Cons	Suitable
Collaborative filtering	Based on similarity to other users	Effective in environments with rich user interaction data; highly personalized recommendations	Large amount of user data	Not suitable for individual prosumers with sensitive data
Content-based filtering	Based solely on the content (features) of the items, historical preferences rather than user interactions	It can handle new items more effectively than collaborative filtering. These systems can handle new items better	Less personalization compared to collaborative filtering; laborious in comparison to zero-shot text classifier.	Yes, it uses item features to make predictions, which could be somewhat similar to using a zero-shot classifier.
Hybrid systems	Combine collaborative and content-based filtering	Balance advantages of both content-based and collaborative filtering	Require complexity in implementation and integration of different recommendation logic	Not suitable for prosumers as user data interactions are not considered.
Knowledge-based systems	Rule-based systems that do not require user data history	Effective where user preferences are known	Extensive domain knowledge and manual rule setup	Similar to zero-shot learning in that it can make inferences without direct previous examples.
Utility-based recommenders	Computation of the utility of each item for a user	Highly customizable and adaptable to different user specifications	Involve complex calculations, a detailed model of user preferences and item utilities	Zero-shot classifiers could be used to estimate utilities.

**Table 7:** Metrics comparison of two recommendation systems.

Performance Metric	Zero-Shot Classifier	Content-Based Filtering
Self-Sustainability Index (SSI)	85%	74%
Self-Consumption Index (SCI)	91%	77%
Grid Dependence Index (GDI)	15%	22%
Economic Savings Index (ESI)	43%	18%

## Data Availability

The data will be made available upon reasonable request.
